# Evaluating the Role of Vitamin D in Alleviating Chronic Pruritus: A Meta-Analysis

**DOI:** 10.3390/ijms25189983

**Published:** 2024-09-16

**Authors:** Chen-Pi Li, Shin-Chuan Huang, Yao Hsiao, Ru-Yin Tsai

**Affiliations:** 1 Department of Nursing, Tung’s Taichung MetroHarbor Hospital, Taichung 43503, Taiwan; t8369@ms.sltung.com.tw (C.-P.L.); chuan913@yahoo.com.tw (S.-C.H.); 2 Department of Nursing, National Taichung University of Science and Technology, Taichung 40343, Taiwan; 3 School of Medicine, Chung Shan Medical University, Taichung 40201, Taiwan; s1001085@gm.csmu.edu.tw; 4 Department of Anatomy, School of Medicine, Chung Shan Medical University, Taichung 40201, Taiwan; 5 Department of Medical Education, Chung Shan Medical University Hospital, Taichung 40201, Taiwan

**Keywords:** itch management, immunomodulation, cytokine, inflammation, skin lesion area

## Abstract

Chronic pruritus is a distressing condition that significantly impacts patients’ quality of life. Recent research has increasingly focused on the potential role of vitamin D, given its immunomodulatory properties, in managing this condition. This meta-analysis seeks to systematically assess the effectiveness of vitamin D supplementation in alleviating chronic pruritus across diverse clinical contexts. We conducted an extensive search through multiple databases, covering literature up to July 2024, to identify relevant randomized controlled trials (RCTs) that evaluated the effect of vitamin D on chronic pruritus. Eligible studies were those that provided data on changes in pruritus severity, as measured by standardized tools, before and after vitamin D treatment. The data were synthesized using a random-effects model to address variability among the studies. This meta-analysis is registered with PROSPERO (registration number: CRD42024579353). The findings indicate that vitamin D supplementation is associated with a significant reduction in pruritus severity, the skin lesion area, and levels of inflammatory cytokines, including tumor necrosis factor (TNF), interleukin-6 (IL-6), and high-sensitivity C-reactive protein (hs-CRP), compared to controls. These results suggest that vitamin D could be a promising therapeutic option for chronic pruritus, though further rigorous studies are required to validate these findings and to elucidate the mechanisms involved.

## 1. Introduction

Chronic pruritus, a common dermatological condition, can arise from a wide variety of causes. It is particularly prevalent in aging populations, where xerosis (dry skin) plays a significant role due to the reduced barrier function of the skin [1]. Other contributing factors include systemic conditions like chronic kidney disease [2,3] and liver disorders [4]. Chronic pruritus, characterized by persistent itching lasting more than six weeks, is a common and often debilitating symptom associated with various dermatological and systemic conditions. It can significantly impair quality of life, leading to sleep disturbances, psychological distress, and even social isolation [5,6]. Despite its prevalence, the underlying mechanisms of chronic pruritus are complex and not fully understood, making it challenging to manage effectively [7].

Recent studies have suggested that vitamin D, a secosteroid hormone traditionally known for its role in calcium homeostasis and bone health, may have broader immunomodulatory effects that could be beneficial in managing chronic inflammatory conditions, including pruritus [8,9]. The role of vitamin D in regulating immune function is crucial for its antipruritic effects. By reducing the production of pro-inflammatory cytokines, such as IL-6, TNF-α, and IL-17, vitamin D helps alleviate inflammation, a known trigger for chronic pruritus [10]. Additionally, vitamin D enhances the skin barrier by regulating keratinocyte proliferation and differentiation while also promoting the production of antimicrobial peptides such as cathelicidins [11]. Moreover, vitamin D receptors are expressed in various skin cells, including keratinocytes and immune cells, where they influence cell proliferation, differentiation, and cytokine production [12]. These findings have led to growing interest in the potential therapeutic role of vitamin D supplementation for chronic pruritus.

Given that vitamin D deficiency is recognized as a risk factor for numerous diseases, monitoring the serum levels of 25-hydroxy vitamin D [25(OH)D] is crucial. Different health organizations offer varying definitions of deficiency and sufficiency. The Endocrine Society defines vitamin D deficiency as serum 25(OH)D levels below 20 ng/mL, insufficiency as levels between 21 and 29 ng/mL, and sufficiency as levels exceeding 30 ng/mL [8]. The World Health Organization (WHO) aligns with the Institute of Medicine’s (IOM) guidelines, categorizing levels below 20 ng/mL as deficient [13]. These variations highlight the importance of standardizing measurements based on specific patient populations and clinical contexts.

While some observational studies and clinical trials have reported that vitamin D supplementation can reduce the severity of pruritus and improve skin health [14,15], other studies have shown mixed or inconclusive results [16,17]. Given the heterogeneity of existing research and the varied methodologies employed, a systematic evaluation of the evidence is needed to determine whether vitamin D supplementation can be recommended as a reliable treatment for chronic pruritus. This meta-analysis aims to systematically review and synthesize the available evidence on the efficacy of vitamin D supplementation in reducing chronic pruritus severity across different patient populations. By pooling data from multiple studies, we seek to provide a clearer understanding of the potential benefits of vitamin D in this context and identify areas where further research is needed.

## 2. Results

### 2.1. Study Search and Characteristics of Included Patients

**Figure 1:**
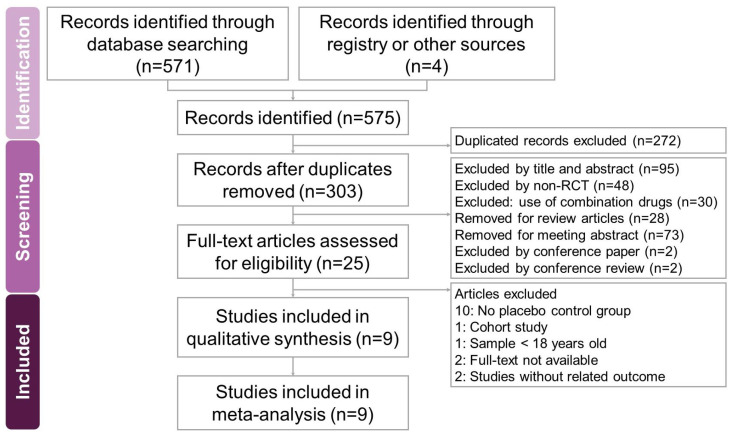
A flowchart illustrating the study selection process for the systematic review and meta-analysis on the effects of vitamin D in reducing chronic pruritus in adults with various dermatological conditions. Out of 575 identified records, only 9 met the eligibility criteria and were included in the final review.

The search and selection process for the trials yielded a comprehensive set of studies for inclusion in this meta-analysis (Figure 1). Our initial search across four databases—PubMed, Embase, Cochrane Library, and Web of Science—along with an additional search using PubMed’s ‘related articles’ feature, yielded a total of 575 trials. After removing duplicates, 303 unique trials remained and were subjected to title and abstract screening, resulting in the exclusion of 278 trials. A detailed full-text review of the 25 remaining trials led to the exclusion of 16 trials for reasons including the absence of a placebo control group (10 trials [18,19,20,21,22,23,24,25,26,27]), being a cohort study (1 trial [28]), involving participants under 18 years old (1 trial [29]), the lack of full-text availability (2 trials [30,31]), and outcomes unrelated to the study’s focus (2 trials [32,33]). Ultimately, nine trials [2,3,15,17,34,35,36,37,38] met the inclusion criteria and were included in this meta-analysis. All selected studies were published in English. Table 1 summarizes the key characteristics of the included trials, which were published between 2004 and 2020. These six trials involved a total of 1085 participants, with the number of participants per trial ranging from 11 to 273. The primary objective of all included trials was to assess the potential effects of vitamin D on chronic pruritus.

### 2.2. Quality Assessment

**Figure 2:**
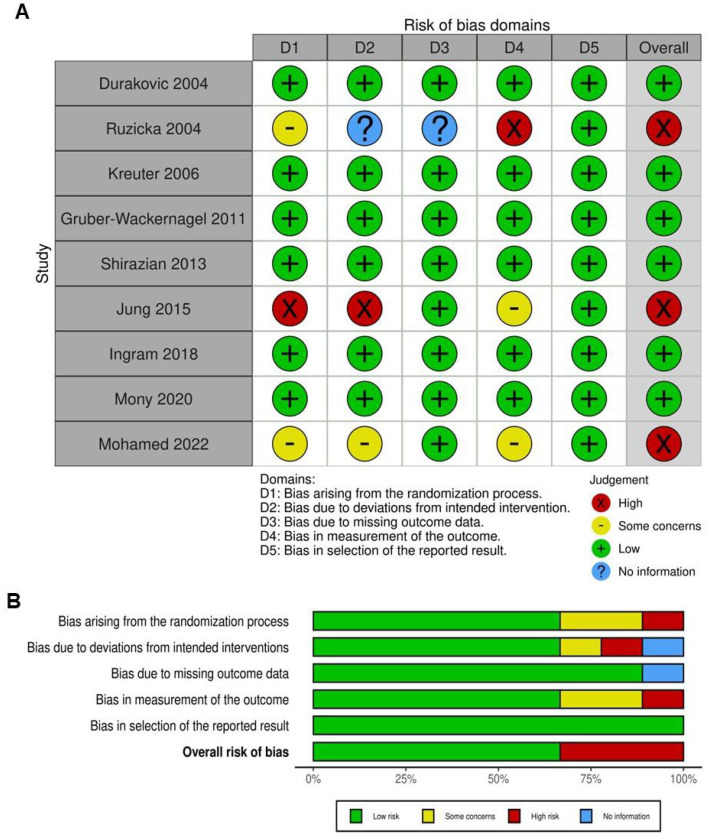
Evaluation of the methodological quality of the included trials. (**A**) Individual risk of bias assessment for each selected study, based on the Rob tool. (**B**) Overall risk of bias summarized as a percentage, considering intention-to-treat and per-protocol analyses. The primary sources of high risk of bias across the studies were deviations from intended interventions, followed by issues related to missing outcome data and deficiencies in the randomization process [2,3,15,17,34,35,36,37,38].

Most trials were assessed as having a low risk of bias, particularly in the selection of reported outcomes (Figure 2). Regarding the randomization process, most studies indicated a low risk of bias, suggesting that randomization was generally well executed. However, there were concerns or insufficient information in some studies [2,36,38], such as Ruzicka (2004), Jung (2015), and Mohamed (2022), leading to uncertainties in this domain. In terms of deviations from intended interventions, most studies also exhibited a low risk of bias, indicating that the interventions were implemented as planned without significant deviations that could have influenced the results. Nevertheless, Jung (2015), Ruzicka (2004), and Mohamed (2022) presented a higher risk of bias in this area, potentially impacting their outcomes. For the domain of missing outcome data, there was variability, with Jung (2015) displaying a high risk of bias due to incomplete data. Other studies predominantly showed low risk or raised some concerns, indicating that the majority of outcomes were sufficiently reported. When evaluating the measurement of outcomes, most studies demonstrated a low risk of bias. However, there were some concerns in studies like Ruzicka (2004), Jung (2015), and Mohamed (2022), possibly due to issues with the assessment or recording of outcomes. Overall, studies such as Ruzicka (2004), Jung (2015), and Mohamed (2022) were identified with a high risk of bias across multiple domains, which may undermine the validity of their results. Conversely, some studies [15,17,34,37] were rated with a low overall risk of bias, indicating that their findings are more likely to be robust and reliable. In conclusion, while most studies demonstrated a low risk of bias across key domains, certain studies with high or uncertain risks in multiple areas require careful interpretation when considering their results in the broader context of the analysis.

### 2.3. Impact of Vitamin D on Chronic Pruritus

**Figure 3:**
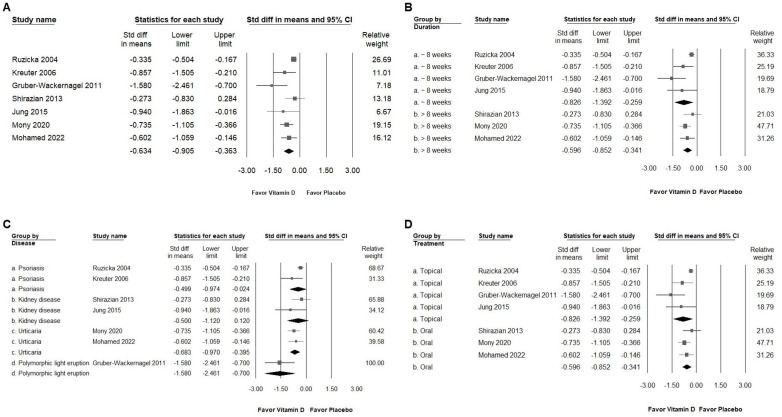
(**A**) displays the overall impact of vitamin D on chronic pruritus, as measured by the visual analog scale, compared to the placebo. (**B**–**D**) show subgroup analyses corresponding to (**A**): (**B**) examines the effect based on the duration of treatment, (**C**) focuses on the specific disease diagnosis, and (**D**) considers the method of treatment administration. The pruritus-relieving effect of vitamin D is represented by squares, which indicate the standardized mean difference, with the squares shifting to the left to signify a reduction in pruritus. The horizontal lines through the squares depict the 95% confidence intervals, while the diamond symbol represents the pooled effect size [2,3,15,17,34,35,36,37,38].

As depicted in Figure 3A, the intervention showed a moderate effect in reducing pruritus among patients who experienced itching (SMD: −0.634, 95% CI: −0.905 to −0.363; *I*^2^ = 56.179%, *p* = 0.033). Subgroup analysis revealed a substantial reduction in pruritus (Figure 3B) for those receiving vitamin D compared to the placebo, particularly in the group with an intervention duration of less than 8 weeks (SMD: −0.826, 95% CI: −1.392 to −0.259; *I*^2^ = 71.683%, *p* = 0.014). In contrast, the group with an intervention duration of more than 8 weeks demonstrated a moderate effect (SMD: −0.596, 95% CI: −0.852 to −0.341; *I*^2^ = 0%, *p* = 0.398). When analyzed by disease diagnosis (Figure 3C), vitamin D exhibited a moderate effect in reducing itch in patients with psoriasis (SMD: −0.499, 95% CI: −0.974 to −0.024; *I*^2^ = 57.198%, *p* = 0.126), kidney disease (SMD: −0.500, 95% CI: −1.120 to −0.120; *I*^2^ = 31.929%, *p* = 0.225), and urticaria (SMD: −0.683, 95% CI: −0.970 to −0.395; *I*^2^ = 0%, *p* = 0.658), whereas a significant effect was observed in the polymorphic light eruption group (SMD: −1.580, 95% CI: −2.461 to −0.700; *I*^2^ = 0%, *p* = 1). Regarding the mode of intervention (Figure 3D), vitamin D applied topically had a significant impact in reducing itch compared to the placebo (SMD: −0.826, 95% CI: −1.392 to −0.259; *I*^2^ = 71.683%, *p* = 0.014), while the oral administration of vitamin D also showed a moderate effect (SMD: −0.596, 95% CI: −0.852 to −0.341; *I*^2^ = 0%, *p* = 0.398). These findings suggest that the topical application of vitamin D may offer more pronounced relief from pruritus compared to oral supplementation.

### 2.4. Effect of Vitamin D on Lesion Area Reduction and Inhibition of Inflammatory Cytokines

**Figure 4:**
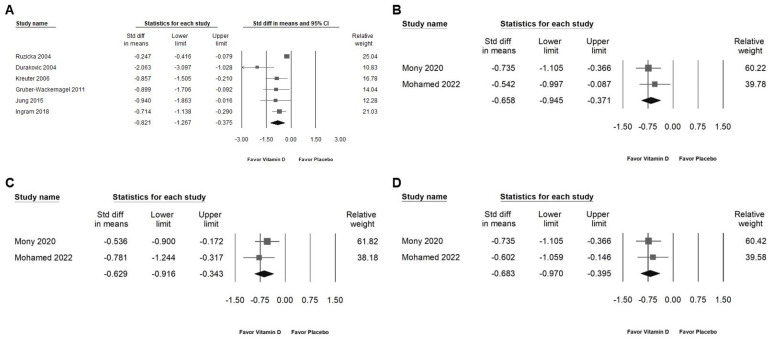
Presents a forest plot that highlights the effects of vitamin D supplementation. The plot is divided into four sections for ease of interpretation: (**A**) shows the effect on the skin lesion area, (**B**) depicts changes in TNF levels, (**C**) assesses alterations in IL-6 levels, and (**D**) evaluates the impact on hs-CRP. The horizontal lines extending from the squares represent the 95% confidence intervals, while the diamond symbols indicate the overall effect sizes [2,3,15,35,36,37,38].

Vitamin D administration significantly reduced the skin lesion area (Figure 4A; SMD: −0.821, 95% CI: −1.267 to −0.375; *I*^2^ = 74.835%, *p* = 0.001). Additionally, vitamin D demonstrated a moderate effect in suppressing inflammatory cytokines, including TNF (Figure 4B; SMD: −0.658, 95% CI: −0.945 to −0.371; *I*^2^ = 0%, *p* = 0.518), IL-6 (Figure 4C; SMD: −0.629, 95% CI: −0.916 to −0.343; *I*^2^ = 0%, *p* = 0.416), and hs-CRP (Figure 4D; SMD: −0.683, 95% CI: −0.970 to −0.395; *I*^2^ = 0%, *p* = 0.658) [2,3,15,35,36,37].

### 2.5. Sensitivity Analysis

**Figure 5:**
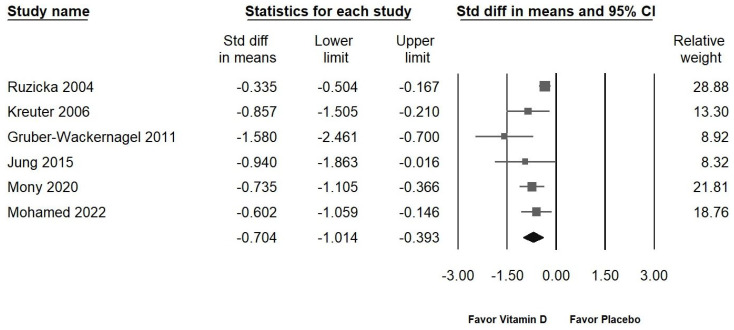
Illustrates the overall effect of vitamin D3 on chronic pruritus, as assessed by the visual analog scale, in comparison to the placebo. The horizontal lines through the squares depict the 95% confidence intervals, while the diamond symbol represents the pooled effect size [2,15,35,36,37,38].

Of the nine studies presented in Figure 3A, one involved the use of vitamin D2 [3], which did not show a significant effect in alleviating chronic pruritus. Consequently, this study was excluded from the sensitivity analysis. Figure 5 demonstrates the impact of vitamin D on reducing chronic pruritus (SMD: −0.704, 95% CI: −1.014 to −0.393; *I*^2^ = 62.027%, *p* = 0.022). The sensitivity analysis revealed no significant differences from the initial findings, confirming that vitamin D continues to exhibit a moderate effect in relieving chronic pruritus.

### 2.6. Publishing Bias

**Figure 6:**
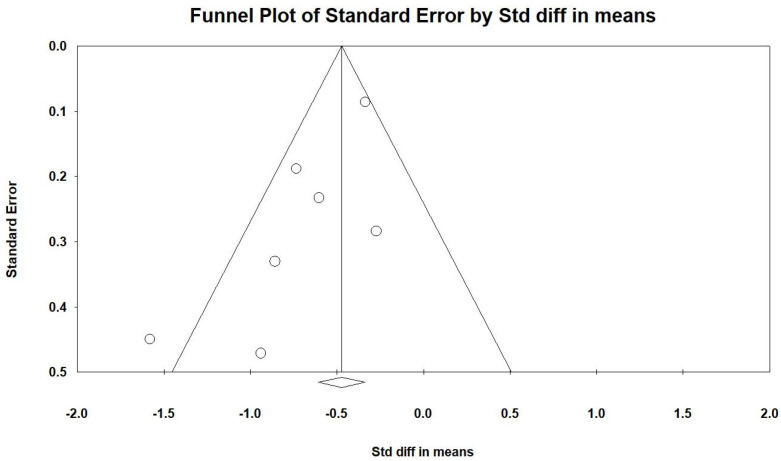
The lines represent the confidence intervals around the effect estimates, indicating the range within which the true effect size is likely to fall. Each circle corresponds to an individual study included in the meta-analysis, with the size of the circle potentially reflecting the study’s weight or sample size. Larger circles denote studies with greater weight or larger sample sizes. The diamond symbol signifies the overall effect estimate from the meta-analysis. The center of the diamond marks the pooled effect size, and the width of the diamond indicates the confidence interval for this estimate [2,3,15,35,36,37,38].

Egger’s regression analysis indicated significant publication bias in our dataset (*p* = 0.03451). Figure 6 displays the funnel plots, which show the SMD for the efficacy of vitamin D. The symmetry of the plot suggests that significant publication bias is present. The noticeable empty space in the lower right corner of the funnel plot raises important concerns. This gap may indicate the existence of unpublished or undiscovered studies with less significant effects of vitamin D, which could impact the overall interpretation of the meta-analysis results.

## 3. Discussion

This meta-analysis examined the effectiveness of vitamin D in managing chronic pruritus across diverse patient populations and intervention methods. The results suggest that vitamin D supplementation, especially in its D3 form, may significantly reduce the severity of chronic pruritus. This effect was found to be more pronounced with topical administration compared to oral intake. Furthermore, the use of vitamin D was associated with a reduction in the skin lesion area and the inhibition of inflammatory cytokines such as TNF, IL-6, and hs-CRP. Although only two of the included studies specifically analyzed the anti-inflammatory effects of vitamin D, these findings are consistent with the existing literature, which highlights the immunomodulatory properties of vitamin D that may help alleviate pruritus by modulating inflammatory responses [8,9].

The mechanisms through which vitamin D exerts its pruritus-relieving effects remain an area of active investigation. Vitamin D receptors (VDRs) are expressed in various skin cells, including keratinocytes, melanocytes, and immune cells such as T lymphocytes and dendritic cells [8]. Upon activation, VDRs influence the expression of numerous genes involved in skin barrier function, immune modulation, and inflammation. Vitamin D has been shown to downregulate the production of pro-inflammatory cytokines, such as TNF-α, IL-6, and hs-CRP, which are often elevated in conditions associated with chronic pruritus [39]. The observed reduction in pruritus severity in our meta-analysis may be attributable to these anti-inflammatory effects, suggesting that vitamin D could be particularly beneficial for patients with inflammatory skin conditions.

Our analysis identified a notable difference in the efficacy of vitamin D2 versus D3 in reducing pruritus. The one study that utilized vitamin D2 reported less significant effects compared to those using D3, which may be due to differences in the metabolic pathways and biological potency of these two forms of vitamin D [40]. Vitamin D3 (cholecalciferol) is more effective at raising serum 25-hydroxyvitamin D levels and has a longer duration of action compared to vitamin D2 (ergocalciferol) [41], which might explain the observed differences in pruritus alleviation. This finding underscores the importance of standardizing the form of vitamin D used in future studies to ensure the consistency and comparability of results. The clinical implications of these findings are potentially significant. Vitamin D, particularly in its D3 form, could be considered as an adjunctive therapy for patients suffering from chronic pruritus, especially in cases where conventional treatments have failed. The topical application of vitamin D appears to offer a more direct and effective route for managing skin-related symptoms, likely due to higher local concentrations of the active compound at the site of inflammation. Therefore, clinicians should be mindful of the form and dosage of vitamin D, as well as the specific patient population being treated, to optimize therapeutic outcomes.

Our analysis also revealed that shorter intervention durations (less than 8 weeks) were associated with a more pronounced reduction in pruritus severity compared to longer interventions. While there is no strong evidence that the long-term use of vitamin D results in a complete loss of effectiveness, the initial benefits might diminish once adequate levels are reached [8]. However, the limited number of studies and the variation in chronic pruritus etiology among the included trials suggest that this finding should be interpreted with caution. It raises an important question about the optimal duration of vitamin D therapy for pruritus, warranting further research. It is essential to monitor vitamin D levels and consult with a healthcare provider to ensure that long-term supplementation remains beneficial and safe.

The fact that most randomized controlled trials (RCTs) on psoriasis pruritus involve the use of vitamin D in combination with other medications reflects the common clinical practice of using combination therapy to achieve better outcomes [42,43,44]. However, this approach complicates the ability to isolate the specific effects of vitamin D alone on the pruritus and lesion area. Given that only four studies [15,17,34,38] included in this meta-analysis focus on the use of vitamin D as a monotherapy for psoriasis, it becomes challenging to draw definitive conclusions about its efficacy in this context. The limited number of studies may reflect a gap in the literature where the specific role of vitamin D alone is underexplored. This could be due to the prevailing view that vitamin D’s effects might be enhanced when used alongside other treatments, such as corticosteroids or other immunomodulators. The inclusion of only four studies on psoriasis limits the generalizability of the findings related to this condition. Clinicians should be cautious when applying these results to practice, as the effects observed in combination therapies may not fully reflect the potential of vitamin D monotherapy. The scarcity of studies investigating vitamin D monotherapy for psoriasis pruritus suggests an area where further research is needed. Future RCTs could focus on evaluating the standalone efficacy of vitamin D in managing psoriasis symptoms, particularly pruritus and lesion reduction. Such studies would be valuable in understanding the specific contribution of vitamin D to psoriasis management and could help in developing more targeted treatment strategies.

This meta-analysis has several limitations that should be acknowledged. First, the heterogeneity among the included studies, particularly in terms of the study design, inter-vention duration, and vitamin D dosage, may have influenced the overall outcomes. Although a random-effects model was used to account for this variability, the inherent differences between studies could still introduce bias. Our findings suggest that shorter intervention durations may be more effective than longer ones; however, this observation may be influenced by the limited number of studies included. Second, the small sample sizes in most of the studies may limit the generalizability of the results, as smaller studies often lack the statistical power to detect significant effects. Third, excluding studies that were not available as full-text papers may have introduced publication bias, potentially overlooking relevant data published in other formats. Fourth, the inclusion of RCTs from Germany, Austria, Korea, the United States, New Zealand, India, and Egypt enriches the meta-analysis by providing a broad, global perspective on the role of vitamin D in managing chronic pruritus. However, the diversity in populations, healthcare systems, and study designs necessitates careful consideration of potential heterogeneity in the data. It would be beneficial to conduct subgroup analyses, where feasible, to explore whether the effectiveness of vitamin D varies significantly by region or population characteristics. Fifth, seasonal changes play a notable role in the progression of chronic pruritus and psoriasis [45]. Increased exposure to sunlight during the summer months may enhance vitamin D production in the skin, which could help alleviate symptoms of both conditions. In contrast, psoriasis tends to worsen in the winter, likely due to reduced sun exposure, leading to lower serum vitamin D levels and heightened pro-inflammatory cytokine activity. Research also suggests that ultraviolet radiation in the summer can modulate the immune response by shifting it away from pro-inflammatory pathways, such as the Th1/Th17 axis, which is closely associated with psoriasis development. Therefore, it is important to take seasonal factors into account, in addition to vitamin D supplementation, when managing pruritus and psoriasis [45]. Additionally, the variability in methods used to assess chronic pruritus across studies presents a challenge in standardizing outcome measurements, which may affect the consistency of the results. Chronic pruritus can stem from various underlying conditions, and while vitamin D interventions appeared to be effective across the included studies, further high-quality RCTs with larger patient populations are necessary to confirm these findings and to gain a more comprehensive understanding. Despite these limitations, the results of this meta-analysis contribute valuable insights into the potential role of vitamin D in managing chronic pruritus. The findings suggest that vitamin D could be a promising therapeutic option, particularly for patients with conditions that are responsive to its immunomodulatory effects.

## 4. Methods and Materials

### 4.1. Data Sources and Selection Criteria

Our study involved a comprehensive search for randomized controlled trials (RCTs) assessing the impact of vitamin D on chronic pruritus. We systematically explored several databases, including PubMed, Embase, Cochrane Library, and Web of Science, up to July 2024. The search strategy included key terms such as “vitamin D”, “chronic pruritus”, “pruritus”, “itch”, and “chronic itch”, with an emphasis on clinical trials conducted in human subjects. Our approach followed the Preferred Reporting Items for Systematic Reviews and Meta-Analyses (PRISMA) guidelines. In addition to screening the identified articles, we examined their reference lists to find further relevant studies. We limited our inclusion criteria to studies published in English and excluded case reports, technical papers, conference abstracts, reviews, letters, editorials, and laboratory-based research. This meta-analysis has been registered with PROSPERO under the identifier CRD42024579353.

### 4.2. Selection of Studies

Two researchers, C.-P.L. and S.Y.L., independently conducted the screening and selection of studies, with a third researcher, R.-Y.T., providing verification. To ensure a comprehensive analysis, hard copies of all pertinent articles were obtained and carefully reviewed. The details of the study selection process are illustrated in the accompanying PRISMA flow diagram (Figure 1).

### 4.3. Data Extraction

The authors C.-P.L. and S.Y.L. independently conducted data extraction using a standardized template aligned with the protocols outlined in the Cochrane Handbook, specifically following the guideline [46,47]. The data extracted encompassed key details such as the study authors’ names, the year and country of publication, the inclusion criteria for participants, the demographic information (including the number of participants and their ages), the study design, the intervention specifics, as well as the outcomes and the methods used to measure them.

### 4.4. Outcomes

The primary outcome of this study was the evaluation of chronic pruritus. Secondary outcomes included the assessment of the skin lesion area and inflammatory cytokines—specifically, tumor necrosis factor (TNF), interleukin-6 (IL-6), and high-sensitivity C-reactive protein (hs-CRP).

### 4.5. Assessment of Methodological Quality 

The researchers C.-P.L. and S.-C.H. independently evaluated the potential biases in the included studies using the Cochrane Collaboration’s Risk of Bias tool for quality assessment. Any discrepancies in their evaluations were resolved through discussion with a third reviewer, R.-Y.T., to reach a consensus. A study was deemed to have a high risk of bias if it exhibited risks in one or more of the assessed domains.

### 4.6. Statistical Analyses

For each included study, the Standard Mean Difference (SMD) and 95% confidence intervals (CIs) were calculated to assess the differences in outcomes between the intervention and control (placebo) groups. These SMDs were then combined using a random-effects model to account for variability across studies. All statistical analyses were performed using Comprehensive Meta-Analysis software (Version 3.0 Biostat, Englewood, NJ, USA). Study heterogeneity was evaluated using the *I*^2^ statistic, with values exceeding 50% indicating significant heterogeneity. Publication bias was assessed through funnel plots and Egger’s regression test, applying a significance level of *p* < 0.05 for most analyses, except for publication bias, where a threshold of *p* < 0.10 was used. Additionally, subgroup analyses were conducted to identify potential sources of heterogeneity, and sensitivity analyses were performed by systematically excluding individual studies to ensure the robustness of the overall findings.

## 5. Conclusions

This meta-analysis provides evidence that vitamin D supplementation has a moderate and statistically significant effect in reducing chronic pruritus, particularly when administered topically. The findings suggest that vitamin D not only reduces skin lesion areas but also exerts a beneficial influence by inhibiting inflammatory cytokines such as TNF, IL-6, and hs-CRP. Although the overall impact of vitamin D is promising, the differences observed between vitamin D2 and D3, as well as the varying effects depending on the route of administration and duration of treatment, highlight the need for further high-quality randomized controlled trials. These future studies should aim to clarify the optimal form, dosage, and application method of vitamin D to maximize its therapeutic potential in managing chronic pruritus.

## Figures and Tables

**Table 1:** Characteristics of included studies.

Author (year)/Country	Inclusion Criteria	Exclusion Criteria	Sample Size (% of Male)/Age	Study Design	Placebo Using	Intervention/ Duration	Main Results	Secondary Results
~8 weeks								
Ruzicka (2004) [38]/Germany	Male and female patients aged 18 to 79 years with scalp psoriasis.	Patients with elevated calcium or phosphate levels, those who had used topical retinoids or vitamin D3 analogs, patients with severe comorbidities, known hypersensitivity to vitamin D3 analogs, or conditions that could interfere with the assessment of the study medication’s efficacy, safety, or tolerability, as well as women of childbearing potential who were not using effective contraception.	P:273 I: 273 (51.2)/ P: not explicitly detailed I: average age 45 years	RCT/double-blind/placebo	Placebo emulsion	Tacalcitol emulsion (4 µg/g) applied once daily to the scalp/8 weeks	The tacalcitol group showed a significant improvement in scalp psoriasis, with the median sum score decreasing by 53% compared to 30% in the placebo group (*p* < 0.0001). Additionally, tacalcitol was significantly superior to placebo in reducing erythema, scaling, and infiltration.	Tacalcitol also showed a significant improvement in patient-reported outcomes, including itching and scaling. The treatment was well tolerated, with similar incidences of local side effects in both the tacalcitol and placebo groups.
Kreuter (2006) [15]/Germany	Adult patients, at least 18 years old, with continuous intertriginous psoriasis for a minimum of 6 months, and otherwise healthy.	Patients who had used systemic corticosteroids, immunosuppressants, or UV light therapy (such as UV-A, UV-B, or psoralen–UV-A) in the previous 4 weeks, those who had received topical treatment for target lesions in the previous 2 weeks, or those with acute guttate or pustular psoriasis, pregnancy or lactation, severe concurrent infectious diseases, diseases associated with immunosuppression or malignancy, drug dependency, mental dysfunction, or other factors limiting compliance.	P: 20 (60) I: (1% Pimecrolimus): 20 (60) I: (0.005% Calcipotriol): 20 (75) I: (0.1% Betamethaone Valerate): 20 (50)/ P: 53.8 ±17.1 I: (1% Pimecrolimus): 53.2 ±14.5 I: (0.005% Calcipotriol): 52.1 ±13.3 I: (0.1% Betamethaone Valerate): 50.4 ±11.9	RCT/double-blind/placebo/ single-center	Vehicle cream	1% pimecrolimus, 0.005% calcipotriol, 0.1% betamethasone valerate, or vehicle cream/4 weeks	VAS for pruritus: decreased by 78% for 0.1% betamethasone, 57% for 0.005% calcipotriol, 35% for 1% pimecrolimus, and 43% for the vehicle.	After 28 days, the mean reduction in Modified Psoriasis Area and Severity Score (MPAS) was 86.4% for 0.1% betamethasone, 62.4% for 0.005% calcipotriol, 39.7% for 1% pimecrolimus, and 21.1% for the vehicle. Betamethasone was significantly more effective than pimecrolimus and the vehicle (*p* < 0.05
Gruber-Wackernagel (2011) [35]/Austria	Patients aged above 18 years with a confirmed diagnosis of PLE based on typical patient history, histology of skin lesions, or positive photoprovo-cation results.	Patients with a presence or history of malignant skin tumors, dysplastic nevus syndrome, photosensitive diseases such as porphyria, chronic actinic dermatitis, xeroderma pigmentosum, or basal cell nevus syndrome, and autoimmune disorders such as lupus erythematosus or dermatomyositis were excluded from the study. Additional exclusion criteria included psychiatric disorders, immune deficiency, or systemic treatment with steroids and/or other immunosuppressive drugs within 6 months before the study. Pregnancy, lactation, UV exposure in the test fields within 8 weeks before the study, general poor health status, severe liver or renal disease, and disorders of calcium metabolism or therapy for such disorders with vitamin D-containing drugs were also exclusion factors.	1. 13 patients (3 men, 10 women; 23% male). 2. Mean age: 37.4 years	RCT/double-blind/placebo/ intraindividual half-body trial	Placebo cream	Calcipotriol cream applied twice daily for 7 days before the start of photoprovocation testing with solar-simulated UV radiation.	Calcipotriol pretreatment significantly reduced PLE symptoms by an average of 32% compared with the placebo throughout the observation period from 48 to 144 h after the first photoprovocation exposure (*p* = 0.0022)	Calcipotriol pretreatment resulted in a significantly lower PLE test score in 58% (48 h), 75% (72 h), and 83% (144 h) of the cases compared with the placebo. Reduced erythema and increased pigmentation were observed with calcipotriol pretreatment.
Jung (2015) [2]/Korea	Patients with chronic kidney disease-associated pruritus (CKD-aP) who were undergoing hemodialysis.	Not explicitly mentioned in the provided text.	P:10 (30) I:10 (30)/Mean age: 63.3 years	RCT/double-blind/placebo/ single-center, open-label pilot study	Vehicle solution	Topical calcipotriol or vehicle solution applied twice daily for 1 month	Both the MPAS and VAS scores significantly decreased after 2 and 4 weeks of topical vitamin D treatment compared to the vehicle (*p* < 0.05). Additionally, dermoscopic evaluation showed significant improvement in skin dryness in the vitamin D-treated group compared to the vehicle group.	No significant side effects were observed.
>8 weeks								
Durakovic (2004) [34]/United States	Patients with moderate plaque psoriasis involving at least 5% of body surface area, two target lesions of at least 5 cm in diameter, and plaque elevation, scaling, and erythema with at least moderate severity (a score of 2 on a scale of 0–4).	Patients with a history of hepatic failure, renal failure, nephrocalcinosis, hypercalcemia, hypercalciuria, or hyperphosphatemia were excluded from the study, as were women of childbearing age who were pregnant, lactating, or unwilling to use effective contraception. Additionally, patients using calcium supplements or drugs that influence calcium metabolism were also excluded.	Paricalcitol-treated Group: 11 (88), mean age 46.5 years (range 29–65).	RCT/double-blind/placebo/self-controlled study	Placebo ointment	15 µg/g paricalcitol ointment (19-nor-1α,25-dihydroxyvitamin D2) once daily/12 weeks	Paricalcitol-treated lesions showed a significant decrease in scaling (74%), erythema (69%), and plaque elevation (71%) compared to placebo-treated lesions, which showed reductions of 32%, 22%, and 8%, respectively.	Serum calcium and phosphorus levels, as well as the 24-hour urinary calcium/creatinine ratio, remained within normal ranges. Immunohistochemical analysis showed that paricalcitol treatment markedly reduced the immunoreactivity of transglutaminase K in psoriatic lesions, bringing it closer to the pattern observed in non-lesional skin.
Shirazian (2013) [3]/United States	Adult patients undergoing maintenance HD who described excessive pruritus and had been on HD for more than 3 months.	With PTH levels less than 70 pg/mL or greater than 1000 pg/mL, serum phosphorus levels greater than 7.0 mg/dL, or serum calcium levels greater than 11 mg/dL were excluded from the study. Other exclusion criteria included the presence of active malignancy and current treatment with ergocalciferol.	P:25 (56) I:25 (60)/ P: 66.2 ± 13.7 I: 66.1 ± 14.7	RCT/double-blind/placebo	Placebo pill	Ergocalciferol 50,000 international units (IU) or placebo once weekly for 12 weeks	Both groups experienced a decrease in pruritus scores, with a reduction of 38.9% in the treatment group and 47.5% in the placebo group. The treatment*time interaction was not statistically significant (*p* = 0.34), indicating no significant difference in pruritus scores between the treatment and placebo groups.	There were no significant differences in calcium, phosphorus, and PTH levels between the groups. However, there was a significant increase in 25-hydroxy vitamin D levels in the treatment group compared to the placebo group (19 ng/mL vs. 1.4 ng/ mL, *p* < 0.01).
Ingram (2018) [17]/New Zealand	Adults aged 18 years and older with chronic plaque psoriasis, who had stable psoriasis not requiring systemic treatment and no history of using more than 1000 IU/day of vitamin D supplements in the last two months.	Patients with chronic kidney or liver disease, those who smoked, were pregnant, lactating, or planning a pregnancy were excluded from the study. Additionally, the use of phototherapy, systemic steroids, or other psoriasis treatments within the last three months was also an exclusion criterion.	P:34 (50) I:67 (58)/ P: 46.7 ± 13.7 I: 50.7 ± 13.44	RCT/double-blind/placebo	Placebo capsules identical in appearance to vitamin D3 capsules, taken once monthly for 12 months.	Monthly oral doses of 100,000 IU of vitamin D3.	There was no significant difference in PASI scores between the vitamin D and placebo groups over the 12-month period. Both groups showed a mild improvement in PASI scores from baseline, but the improvement was not significantly different between the groups.	Serum 25(OH)D concentrations significantly increased in the treatment group and unexpectedly also increased in the placebo group, possibly due to increased sun exposure or other factors.
Mony (2020) [37]/India	Adults aged 20 to 50 years with CU for more than 6 weeks and vitamin D deficiency (serum vitamin D < 20 ng/mL) were included in the study.	Patients with acute urticaria, physical urticaria, urticarial vasculitis, hereditary or acquired angioedema, symptoms of vitamin D deficiency such as musculoskeletal pain or fractures, hepatic or renal dysfunction, malignancies, infections, or inflammatory cutaneous disorders were excluded from the study. Pregnant and lactating women, as well as patients who had taken vitamin D supplementation in the past 6 months were also exclusion.	P:60 (21) I:60 (20)/ P: 36.71 ± 11.01 I: 38.80 ± 12.54	RCT/double-blind/placebo	Matched placebo	Experimental group: 60,000 IU of vitamin D3 (cholecalciferol) fortnightly for 12 weeks. Control group: similar-looking placebo fortnightly for 12 weeks. Both groups received standard treatment with levocetirizine.	There was a significant reduction in UAS7 and medication dosage in the vitamin D-treated group compared to the placebo group (*p* < 0.0001). Additionally, there was a significant reduction in inflammatory cytokines, IL-6, IL-17, TGF-β, and hs-CRP, in the vitamin D-treated group compared to the placebo group.	There was a significant increase in 25-OH vitamin D and vitamin D binding protein levels in the vitamin D-treated group compared to the placebo group. In contrast, there was no significant change in cytokine concentrations or vitamin D levels in the placebo group.
Mohamed (2022) [36]/Egypt	Adults > 18 years of age, having urticaria episodes at least 2 days per week for 6 weeks or longer with/without angioedema	Patients with only physical urticaria, urticarial vasculitis, hereditary or acquired angioedema were excluded, as were those with dyslipidemia, diabetes, hypertension, pre-existing cardiovascular disease, cerebrovascular accidents, hypothyroidism, smokers, and other systemic or cutaneous disorders such as atopic dermatitis or psoriasis. Additionally, patients with hypercalcemia (>11 mg/dL), diabetes, renal insufficiency, hepatic disorders, hyperparathyroidism, sarcoidosis, other granulomatous disorders, or malignancy were excluded. Pregnant and lactating women, as well as those who had taken vitamin D supplementation in the past 6 months were also exclusion.	P:67 (50.7) I:77 (40.2)/ P: 39.34 ± 7.23 I: 36.50 ± 5.12	RCT/single-blind/placebo	Matched placebo	Study group: received 0.25 µg alfacalcidol once daily for 12 weeks in addition to the standard therapy (Hydroxyzine 25 mg/day). Placebo group: received an oral placebo taken with the same regimen for 12 weeks in addition to the standard therapy (Hydroxyzine 25 mg/day)	The UAS7 total score was significantly lower in the study group after active vitamin D administration compared to the placebo group (*p* < 0.01)	No significant change in the UAS7 total score or the number of patients in each severity level in the placebo group compared to their baseline results. In the study group, there was a significant increase in mean serum [25(OH) D] levels compared to the placebo group and their baseline results (*p* < 0.001). Additionally, the study group showed a significant decrease in mean serum IL-6, hs-CRP, and TNF-α levels compared to the placebo group and their baseline results (*p* < 0.01). Moreover, there was a significant negative correlation (r = −0.67, *p* < 0.05) between serum [25(OH) D] levels and total UAS7 scores, indicating disease severity.

AD: atopic dermatitis; Aer: aerosol; CU: chronic urticarial; HD: hemodialysis; I: intervention; MPAS: modified psoriasis area and severity index; P: placebo; PASI: psoriasis area and severity index; PTH: parathyroid hormone; PLE: polymorphic light eruption; USA7: urticaria activity score over 7 days; VAS: visual analog scale.

## Data Availability

All data generated or analyzed during this study are included in this published article.
